# Analytical Considerations of Stable Isotope Labelling in Lipidomics

**DOI:** 10.3390/biom8040151

**Published:** 2018-11-16

**Authors:** Alexander Triebl, Markus R. Wenk

**Affiliations:** Department of Biochemistry, Yong Loo Lin School of Medicine, National University of Singapore; Singapore 117596, Singapore; bchmrw@nus.edu.sg

**Keywords:** lipidomics, stable isotope labelling, mass spectrometry, flux analysis

## Abstract

Over the last two decades, lipids have come to be understood as far more than merely components of cellular membranes and forms of energy storage, and are now also being implicated to play important roles in a variety of diseases, with lipid biomarker research one of the most widespread applications of lipidomic techniques both in research and in clinical settings. Stable isotope labelling has become a staple technique in the analysis of small molecule metabolism and dynamics, as it is the only experimental setup by which biosynthesis, remodelling and degradation of biomolecules can be directly measured. Using state-of-the-art analytical technologies such as chromatography-coupled high resolution tandem mass spectrometry, the stable isotope label can be precisely localized and quantified within the biomolecules. The application of stable isotope labelling to lipidomics is however complicated by the diversity of lipids and the complexity of the necessary data analysis. This article discusses key experimental aspects of stable isotope labelling in the field of mass spectrometry-based lipidomics, summarizes current applications and provides an outlook on future developments and potential.

## 1. Introduction

Lipidomics is the study of cellular lipids and their roles in health and disease [1]. The field came to prominence in the early 2000s [2,3] and has now become indispensable in basic and clinical research, as lipids emerge more and more from being viewed as merely energy-storage molecules, and instead are being implicated in a plethora of pathological conditions, such as metabolic syndrome, cardiovascular and neurodegenerative diseases, as well as cancer [4].

Most lipidomic studies are based on the determination of the levels of lipids and on their comparison either between disease and control groups, at different time points, or before and after treatments. This is by design merely a static measurement of what is really a very dynamic metabolism, and whenever, e.g., differences in the concentrations of circulating lipids in plasma are found, this might either be due to different rates of synthesis or degradation, or due to the release from another storage pool. For example, an increase of plasma triacylglycerols may be due to uptake from food or to release from adipose tissue. While this difference is impossible to assess using pure concentration-based approaches, it may have important different biochemical implications. While the absolute concentration of phosphatidic acid is constantly low, it still has a high rate of metabolic flux, and due to its central position in phospholipid metabolic pathways, is constantly biosynthesized and processed into other phospholipids [5].

Therefore, when it comes to investigating lipid metabolism and dynamics-biosynthesis directly, transport, interconversion and degradation-stable isotope labelling is the technique of choice and provides an additional dimension of information to absolute quantitative values. In all stable isotope labelling studies, a stable isotope label in a substrate, usually ^13^C or deuterium (^2^H), is metabolized into all resulting metabolites [6]. Stable isotope tracing therefore enables the determination of the metabolic fate of stable isotope-labelled precursors. This approach has been used since as early as the 1930s, when deuterium labelling was first used to investigate murine fatty acid metabolism and determine that even under a hypocaloric diet, dietary fatty acids were not immediately oxidized, but stored and released from fat tissue [7]. Far before the age of bioanalytical mass spectrometry, the deuterium content was measured using refractometry and densitometry in water after combustion of the labelled biomolecules.

Nowadays, modern instrumentation offers a far greater level of detail. Coupling of chromatography and mass spectrometry enables more than just the determination of total labelling; instead, labelling of individual lipid species can be investigated, and even the localization and the enrichment of the label within lipids can be determined using appropriate analysis strategies. Stable isotope labelling has already been applied to numerous studies of lipid metabolism (recently reviewed by Ecker and Liebisch [8]), and the techniques used have evolved with time and with the analytical instrumentation available.

This article discusses the fundamentals and important experimental aspects of stable isotope labelling for application to mass spectrometry-based lipidomics and offers an outlook on the future and the potentials of this technique.

## 2. Fundamentals

### 2.1. Stable Isotope-Labelled Compounds in Lipidomics

Stable isotope labelled lipids are routinely used in lipidomic laboratories, yet not as tracers for labelling studies. They are considered ideal internal standards, and the gold standard for absolute quantitation [9,10,11], but the commercial availability of suitable isotope-labelled standards is limited compared to the complexity of the lipidome, which comprises hundreds and thousands of different lipids. Techniques such as lipidome isotope labelling of yeast (LILY) [12] attempt to solve this problem by growing *Pichia pastoris* on uniformly ^13^C-labelled glucose as the carbon source, thereby successively labelling yeast metabolites with ^13^C to a labelling degree of over 99.5%. Obtaining a near-complete labelling efficiency is important, as otherwise, the concentration of lipids would be overestimated. The labelled yeast lipid extract can then be used to correct for matrix effects, both in yeast and other matrices such as human plasma [13], and for more accurate quantitation. Although the yeast lipidome comprises fewer lipid classes and lipid species than more complex mammalian lipidomes, this is a helpful approach to account for ionization differences and matrix effects during lipidomic analysis.

**Figure 1:**
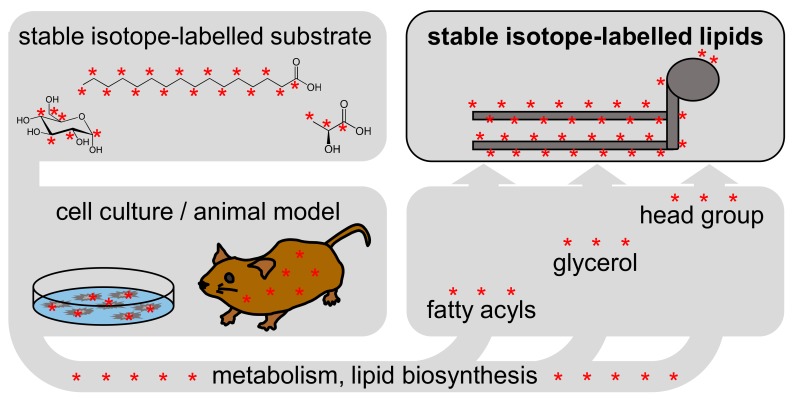
Stable isotope labelling workflow in biological systems. A stable isotope-labelled substrate is metabolized into the lipid building blocks (fatty acyls, glycerol and head group).

The LILY approach includes the fundamental principle of stable isotope labelling, depicted in Figure 1: A stable isotope-labelled substrate is metabolized by a biological system, and the stable isotope substrate is incorporated into the resulting metabolites. As shown schematically in Figure 1, phospholipids consist of fatty acyls, a glycerol backbone, and a head group. Depending on the metabolic pathway of the substrate and the duration of labelling, lipids may be partially or fully labelled, labelled across all, or only on certain building blocks.

### 2.2. Tracers and Tracees

Stable isotope labelling makes use of the simple fact that while isotopes (nuclides with different number of neutrons) have different mass, they behave chemically identical. Therefore, stable isotope-labelled molecules (“tracers”) have the same biochemical properties as their monoisotopic equivalents, and a stable isotope-labelled tracer is metabolized identically to the unlabelled form (the “tracee”) [14]. Tracers and tracees can however be detected separately using analytical techniques such as mass spectrometry or nuclear magnetic resonance (NMR).

Historically, radiolabelled tracers have frequently been used in metabolism research, but they have now largely been replaced by stable isotope-labelled tracers. While radioactive tracers may have the advantage of higher analytical sensitivity, this is somewhat offset by strict legal restrictions on handling and waste disposal, limitations of use of radiolabelled tracers in humans and potential interference with physiological metabolism [8,15,16].

In the context of stable isotope labelling, the term “mass isotopomer” is often used in in literature [8,17,18,19,20,21,22] to refer to what should correctly be called “isotopologue”. International Union of Pure and Applied Chemistry (IUPAC) defines isotopologue as a “molecular entity that differs only in isotopic composition (number of isotopic substitutions), e.g., CH_4_, CH_3_D, CH_2_D_2_*”* [23], whereas an “isotopomer” is defined as “isomers having the same number of each isotopic atom but differing in their positions” [24], a misleading term when it is used in the context of mass spectrometry, which is not capable of determining the location of an individual atom within an ion. Therefore, compounds with different numbers of heavy isotope labels (^13^C/^2^H/^15^N), together with the corresponding monoisotopic molecule, should correctly be called isotopologues.

## 3. Analytical Considerations

### 3.1. Choice of Tracer

The choice of tracer molecule depends largely on the metabolic pathway in question. Labelling with glucose or heavy water will lead to generalized incorporation of the label into a large number of different metabolites, while more pathway-specific tracers can be chosen, e.g., serine or palmitoyl-CoA for the sphingolipid pathway [25], or lipid head groups such choline can be used to investigate biosynthesis of choline-containing lipid classes. Commonly-used labelled substrates for tracing lipid flux are glucose or glycerol [26,27,28], fatty acids [19,29], amino acids [17,27,30], or heavy water (D_2_O) as an unspecific tracer [31,32]. In animal or human studies, the tracer can be a substantial cost factor, as rather large amounts may be required to achieve a sufficient degree of labelling, and because injectable tracers for human studies must be sterile.

The two most commonly-used heavy isotopes used in labelling of biomolecules are ^13^C and deuterium, as carbon and hydrogen are found in all biomolecules. ^15^N can also be used as a tracer, but its use is limited to nitrogen-containing lipids. If feasible, a ^13^C-labelled tracer is often preferred over deuterium for two reasons: firstly, when a deuterium-labelled tracer is used, deuterium exchange can occur in protic solutions (e.g., during storage), which can decrease the grade of labelling, and secondly, deuterium labels on fatty acids may be lost during fatty acid desaturation.

The potential of tracer recycling is another consideration in the choice of tracer. When tracer recycling occurs, the labelled tracer is biosynthesized again in a second round of metabolism, which would lead to overestimation of the actual degree of labelling and to erroneous calculation of synthesis or flux rates [33,34,35].

### 3.2. Sample Preparation

In lipidomics experiments, cells or tissues are typically flash frozen or immersed in cold organic solvents to stop enzymatic reactions, chemical degradation, oxidation, and to preserve a snapshot of the metabolic state [36]. Labelled lipids behave identically to non-labelled lipids, and existing sample preparation methods do not need to be amended for stable isotope labelling experiments. Lipids are typically extracted using either single-phase protein precipitation methods [37] or two-phase partitioning methods using either chloroform [38,39] methyl-*tert*-butyl ether (MTBE) [40] or a heptane/ethyl acetate mixture [41]. For enrichment of particular lipid classes, or for separation of a lipid extract into its constituent lipid classes, solid-phase extraction (SPE) [42] or thin-layer chromatography [43] can be employed. Furthermore, chemical derivatization can be used for enhanced ionization and improved detection of low abundant lipid classes [44,45]. Derivatization and SPE are time-consuming procedures, which are typically only necessary if the analysis is targeted toward extremely low abundant lipid classes, while for most applications, a single-phase precipitation or a two-phase extraction method is sufficient.

### 3.3. Sample Introduction

**Figure 2:**
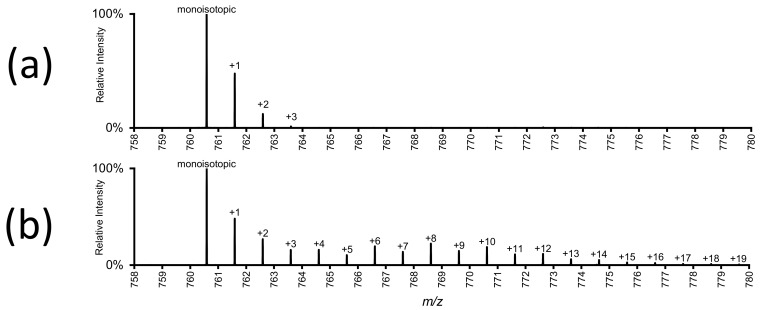
High resolution full-scan mass spectra of a labelled and unlabelled phospholipid. (**a**) PC (phosphatidylcholine) 34:1 from A549 cells grown on non-labelled substrate. The monoisotopic peak at *m*/*z* 760.6, as well as three isotopologues, which represent the natural abundance of ^13^C, are visible. (**b**) Mass spectrum showing the “isotopic envelope” of the same lipid from cells grown on ^13^C_6_-labelled glucose. Varying numbers of ^13^C have been incorporated into the lipid via cellular metabolism. See Figure 4 for these data after correction for natural ^13^C abundance (authors’ unpublished data).

A paradigm decision in all lipidomic analysis is the choice of sample introduction, which can be performed either with chromatographic separation or without (“direct infusion” or “shotgun” “lipidomics”) [46,47]. While chromatographic separation is often the preferred choice for stable isotope experiments, Schuhmann et al. have employed shotgun lipidomics in metabolic labelling experiments [48], labelling cellular lipids with ^15^N-choline and ^15^N-serine, and Sun et al. have used shotgun techniques to analyze a ^13^C_6_ glucose-labelled murine lipidome. The use of the shotgun technique is in these cases possible because high resolution accurate mass spectrometry minimizes the chance of *m*/*z* overlaps. Direct infusion has also been used to probe phospholipid remodelling, using labelled choline as a substrate [49]. Here, the lack of chromatographic separation does not pose a problem, as class-specific precursor ion scans are used, and no complex labelling patterns (as shown in Figure 2) are observed.

With chromatographic separation, an additional selectivity criterion is added, as the labelled molecule must have identical retention time as the unlabelled molecule. While this is usually the case with ^13^C-labelled compounds, deuterium labelling can lead to a considerable retention time shift compared to the monoisotopic compounds, both in normal and reversed phase chromatography [50,51,52] due to different hydrogen bond strengths between protium- and deuterium-containing molecules. In gas chromatography (GC-MS), this effect is less noticeable or not present at all [53].

The metabolism of fatty acids is frequently studied using stable isotope labelling, and analyzed using GC-MS [54,55]. Depending on sample preparation, analysis can be targeted to either free fatty acids (FFA, or non-esterified fatty acids (NEFA)) [53] or to total fatty acids (TFA) comprising both free fatty acids and fatty acids bound to lipids [56]. The advantage of GC is its very high chromatographic resolution, for example for isomeric fatty acids (such as the double bond positional isomers 20:3 ω-3/ω-6/ω-9). However, any metabolic flux analysis of total fatty acids does not give information from which lipid classes or lipid species the fatty acids have originated.

Reversed phase (RP) chromatography is often used for analysis of stable isotope-labelled lipidomes [27,28,57,58,59], and hydrophilic interaction liquid chromatography (HILIC) has also been employed [60]. RP separates lipids according to their overall hydrophobicity (governed largely by hydrocarbon chain length), whereas the separation in HILIC is driven by the lipid head groups, with only minimal separation within individual classes [46]. Therefore, all lipids of one class elute together in HILIC and would produce a very complex spectrum containing all isotopologues of all lipids of one particular class. In reversed phase, however, lipids belonging to the same class, but differing in carbon number and degree of unsaturation, are chromatographically separated, which greatly reduces spectral complexity and simplifies analysis [61].

### 3.4. Instrumental Considerations

Fatty acids can be readily analyzed after derivatization and GC separation by either electron ionization (EI) or chemical ionization (CI) coupled to a single quadrupole mass analyzer. EI produces a spectrum that is dominated by fragment ions [55], whereas CI (after derivatization with pentafluorobenzyl bromide) produces highly abundant molecular ions without further in-source fragmentation and is therefore better suited for isotopologue analysis.

The second paradigm decision in LC-MS analysis is the choice of mass spectrometer. For isotope labelling studies, triple quadrupole instruments or high resolution instruments such as Orbitraps and QTOFs (quadrupole-time-of-flight) are most widely used. Triple quadrupole instruments are best suited for determining the incorporation of labelled building blocks (e.g., of head groups such as labelled choline [49,60] or ethanolamine [62], or of fatty acids [19,57,63]), as these can be readily detected using precursor or neutral loss scans. In contrast, high resolution mass spectrometers rely on the acquisition of full scan spectra with high resolution and high mass accuracy to identify compounds [29,48]. Modern high resolution instruments are routinely capable of resolutions above 100,000 and mass accuracies below 1 ppm, allowing for near-unambiguous determination of isotopologues [26,64]. Another benefit of ultra-high resolution (R > 100,000 [64]) analysis is that it can enable multi-tracer studies, as multiple tracers with different isotopic labels (^13^C or deuterium) and/or different degrees of labelling (^13^C_2_ or ^2^H_2_) can be differentiated from each other by high resolution mass spectrometry [48,65] and therefore be used simultaneously in a biological system.

Depending on the type of analysis, different acquisition modes are preferred. For building block analysis, e.g., incorporation of labelled fatty acids, or of head groups, precursor or neutral loss scans for the labelled building blocks on triple quadrupole instruments are well suited. For analysis of unspecific labelling, e.g., labelling of fatty acids through *de novo* synthesis or elongation, which leads to a much more complex labelling pattern, high resolution accurate mass full scans (such as shown in Figure 2b) provide more information, and allow calculation of the enrichment of the various isotopic states of all lipids detected.

While mass spectrometry is the most widespread technique for analyzing lipid flux, NMR spectrometry is an emerging technique in lipidomics and is already being applied for NMR-based metabolomics [66]. The advantages of NMR are high reproducibility and reliable quantitation, but its applicability for lipid analysis is currently limited by the inability to differentiate between molecular species of one lipid class and its higher detection limit compared to mass spectrometry [67].

## 4. Data Analysis

### 4.1. Information Content

When single-stage mass spectrometry (MS^1^) is used for stable isotope labelling, the isotopic enrichment of the label in individual lipids can be determined. Figure 2b shows the mass spectrum of a labelled phospholipid after reversed phase separation. Using untargeted high-resolution full scanning, other lipids species and all their isotopologues can also be detected, and the isotopic enrichment can be determined for each individual lipid.

Tandem mass spectrometry is typically used in the form of precursor or neutral loss scans for building-block level analysis, such as the incorporation of labelled head groups or of labelled fatty acyls. However, tandem mass spectrometry can also be utilized to determine the location of the isotopic label within a lipid and the isotopic enrichment within lipid building blocks. For example, collisional dissociation of cholesterol esters produces a cholesterol-derived fragment ion. Therefore, if the same mass shift (due to an isotopic label) is observed for both the parent ion and the fragment ion, then the stable isotope label is located not on the fatty acyl, but on the cholesterol moiety [68].

The tandem mass spectrometric behavior of lipids has been extensively studied [69,70,71], and many fragment ions can be obtained which are indicative of the different lipid building blocks. For example, collisional dissociation of phosphatidylcholine in negative ion mode gives rise to multiple ions indicative of the fatty acyls and the head group, and even positional assignment of the fatty acyls is possible by comparing the relative intensities of fatty acyl-derived fragment ions.

**Figure 3:**
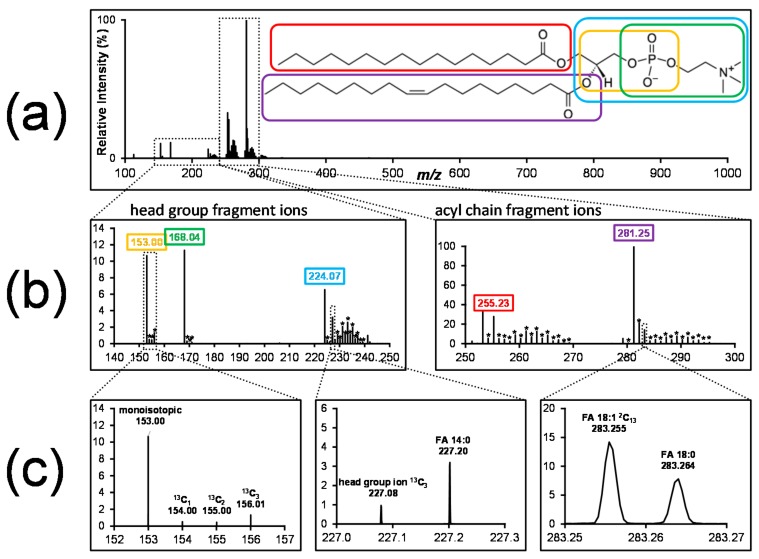
Tandem mass spectrum of stable isotope -labelled PC from A549 cells grown on ^13^C_6_ glucose. (**a**) Tandem mass spectrum of stable isotope-labelled phosphatidylcholine. (**b**) Zoomed to fragment ions indicative of head group (cyclic glycerophosphate, *m*/*z* 153; phosphocholine, *m*/*z* 168; glycerophosphocholine, *m*/*z* 224) and fatty acyls (16:0, *m*/*z* 255 and 18:1, *m*/*z* 281). The isotopologue distributions of individual fragment ions are marked by asterisks. (**c**) Isotopic clusters of individual lipid building blocks (left); high mass resolution is capable of resolving close *m*/*z* values, such as head group fragment ions and fatty acyl ions (middle), as well as resolving signals from fatty acyls with different degrees of labelling (right) (authors’ unpublished data).

Figure 3 shows a product ion spectrum of stable isotope-labelled PC (phosphatidylcholine) and illustrates how high-resolution tandem mass spectrometry can be utilized for detailed labelling analysis. Using appropriate data acquisition methods (i.e., a wide isolation window for fragmentation and sufficient resolution to separate close *m*/*z* values), all isotopologues of one lipid species are selected, fragmented and detected. The resulting fragment spectrum contains fragment ions indicative of the head group and of the fatty acyls, as well as all isotopologues. Similar to the calculation of isotopic enrichment of one lipid species from MS^1^ data, this enables the calculation of isotopic enrichment in the individual lipid building blocks, such as fatty acyls, the glycerol backbone and head group. High mass resolution separates close *m*/*z* pairs belonging to different lipid constituents (Figure 3c, middle panel, ^13^C_3_ glycerophosphocholine, *m*/*z* 227.08, and monoisotopic FA 14:0, *m*/*z* 227.20), or belonging to fatty acyls with different degrees of labelling (Figure 3c, right panel, monoisotopic FA 18:0, *m*/*z* 283.264, and ^13^C_2_ FA 18:1, *m*/*z* 283.255). This illustrates how the information from high resolution tandem mass spectrometry can be utilized to detect the isotopic label in a specific lipid building block.

Tandem mass spectrometry-based approaches have been used to investigate glycosphingolipid turnover [72], cholesterol biosynthesis [68] and alternative phospholipid glycerol biosynthesis pathways in glucose-starved cancer cells [30].

### 4.2. Isotope Correction

Elements used as tracers have naturally-occurring isotopes, which differ in the number of neutrons in the atomic nuclei, and therefore have different atomic mass. Of the two stable isotopes of carbon, ^12^C makes up 98.9% of all carbon atoms, while ^13^C constitutes the remaining 1.1% [73]. Lipids typically contain about 20–60 carbon atoms, which means that the natural abundance of ^13^C already leads to considerable isotopic peaks, even without deliberate labelling (Figure 2a). Any experimentally-induced ^13^C stable isotope labelling is therefore additional to the natural abundance of ^13^C. The same effect is also present for deuterium, even though the natural abundance of deuterium is much lower than that of ^13^C (only 0.01% of hydrogen is deuterium [73]).

Raw mass spectra of stable isotope-labelled compounds, such as shown in Figure 2, need to be corrected for natural isotope distribution. It is important to always include an unlabelled control in the experiment (rather than just adjusting for expected isotope distribution) as the isotopologues of metabolites might not always be measured accurately. Orbitrap mass spectrometers have been shown to underreport the intensities of heavier isotopologues under certain conditions [74], and appropriate experimental control is necessary to recognize this detrimental effect.

**Figure 4:**
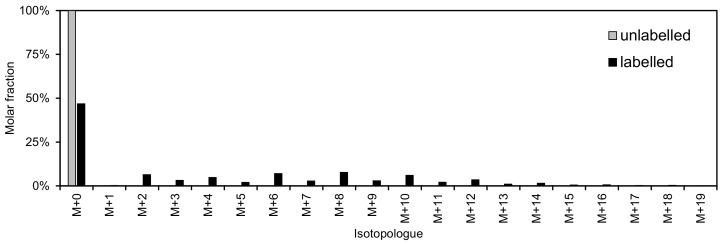
^13^C labelling of PC 34:1 from A549 cells grown on monoisotopic and ^13^C-labelled glucose (data from Figure 2). Isotope correction was performed using IsoCor [75].

Figure 4 shows the data from the spectra in Figure 2 after correction for natural ^13^C abundance with IsoCor [75]: After isotope correction, the cells grown on monoisotopic substrate show no ^13^C labelling, while 53% of PC 34:1 from cells grown on ^13^C_6_-glucose is labelled with one or more ^13^C. The same principles of isotope correction could also be applied to the data from Figure 3 to determine the ^13^C enrichment in the choline head group and the constituent fatty acyls.

There are many freely-available and/or open source software packages for isotope correction, such as IsoCor [75], X13MS [76], AccuCor [74], Isotope Correction Toolbox (ICT) [77] or LS-MIDA [78] (this is a non-exhaustive list and not intended as recommendations). Isotope correction of full-scan data is relatively straightforward, and essentially consists of subtracting the calculated natural abundances of the isotopologues from the measured data based on the elemental composition. Isotope correction of SRM (selected reaction monitoring) data introduces an additional level of complexity, because an isotope effect may or may not be measured, depending on the distribution of the heavy isotopes between the precursor-product ion pairs measured [79].

### 4.3. Flux Analysis

As with most bioanalytical workflows, data analysis of stable isotope labelling experiments is the major bottleneck, in particular since it currently is automated to only a very small degree. Flux analysis is the complex mathematical and statistical treatment of data derived from isotope labelling to quantify metabolic flux. Detailed information about biosynthesized, i.e., labelled, compounds within a biosynthetic pathway is fed into algorithms and modelled on existing knowledge of the metabolic pathways. Flux analysis takes place outside the analytical laboratory and has been described in detail [80,81,82,83,84,85,86,87,88,89,90]. Software packages for flux analysis of small molecules are available (e.g., FiatFlux [91], OpenFLUX [92], 13C-FLUX [93], OpenMoebius [94], INCA [95] or Agilent VistaFlux [96]), but their applicability to integrated lipid flux analysis may be limited.

## 5. Conclusions

Stable isotope labelling is a powerful technique with promising applications. It enables direct analysis of nutrient distribution, metabolism, conversion into metabolites and the fate of the resulting metabolites. In contrast to radioactive labelling, there are no dangers or safety concerns, making this technique particularly well suited for metabolism studies in humans.

Future research using stable isotope labelling in lipidomics will likely make use of ultra-high mass resolution, use multiple tracer methods and utilize the information content of tandem mass spectra to localize and quantify label enrichment within lipid building blocks.

All this combined will allow the metabolism of lipids to be investigated in unprecedented detail, greatly advancing our knowledge of the roles of lipids in health and disease.
